# PSMB2 and RPL32 are suitable denominators to normalize gene expression profiles in bronchoalveolar cells

**DOI:** 10.1186/1471-2199-9-69

**Published:** 2008-07-31

**Authors:** Eva Kriegova, Arsen Arakelyan, Regina Fillerova, Jaromir Zatloukal, Frantisek Mrazek, Zdenka Navratilova, Vitezslav Kolek, Roland M du Bois, Martin Petrek

**Affiliations:** 1 Department of Immunology, Palacky University, The Czech Republic; 2 Department of Respiratory Medicine, Palacky University & Faculty Hospital, Olomouc, The Czech Republic; 3 Interstitial Lung Disease Unit, Royal Brompton Hospital, London, UK

## Abstract

**Background:**

For accuracy of quantitative reverse transcriptase-polymerase chain reaction (qRT-PCR), normalisation with suitable reference genes is required. To date, no reference genes have been validated for expression studies of bronchoalveolar (BAL) cells. The aims of this study were to identify gene(s) with stable mRNA expression in BAL cells irrespective of gender, smoking, BAL cellular composition, lung pathology, treatment; and to assess the influence of reference genes on target gene expression data.

**Results:**

The mRNA expression of ten housekeeping genes (ACTB, ARF1, CANX, G6PD, GAPDH, GPS1, GNB2L1, PSMB2, PSMD2, RPL32) was investigated by qRT-PCR in BAL cells from 71 subjects across a spectrum of lung diseases. The analyses were validated in an independent BAL cohort from 63 sarcoidosis patients and 17 control subjects. A second derivative method was used to calculate expression values (CTt); an equivalence test, applets BestKeeper, geNorm and NormFinder were applied to investigate gene expression stability. Of the investigated genes, PSMB2 (CTt ± SD, 23.66 ± 0.86) and RPL32 (18.65 ± 0.92) were the most stable; both were constantly expressed in BAL samples from parallel investigated cohorts irrespective of evaluated variables. Finally, to demonstrate effect of traditional (ACTB/GAPDH) and novel (PSMB2/RPL32) reference genes as denominators, expression of two cytokines known associated with sarcoidosis was investigated in sarcoid BAL cells. While normalization with PSMB2/RPL32 resulted in elevated IFNG mRNA expression (*p *= 0.004); no change was observed using GAPDH/ACTB (*p *> 0.05). CCL2 mRNA up-regulation was observed only when PSMB2/RPL32 were used as denominators (*p *< 0.03).

**Conclusion:**

PSMB2 and RPL32 are, therefore, suitable reference genes to normalize qRT-PCR in BAL cells in sarcoidosis, and other interstitial lung disease.

## Background

Quantitative reverse transcriptase-polymerase chain reaction (qRT-PCR) has become a method of choice for gene expression studies in clinical samples, especially for low copy targets of interest and for samples of limited size [1-3]. In comparison to microarrays [4], qRT-PCR benefits from broad dynamic range, sensitivity, and enables accurate quantification [5,6].

However, to precisely quantify changes in expression level of target genes by qRT-PCR, one must apply normalisation for heterogeneity in clinical samples and also for variability introduced during RNA extraction and cDNA synthesis [1,7]. Besides normalisation to sample size and total RNA, normalisation using endogenous reference genes represents relevant approach [3]. Reference genes should ideally be constitutively expressed by all cell types and should not be affected by disease and experimental procedure. To date, a universal reference gene has not been identified yet. Housekeeping genes (HKGs) are most commonly used reference genes [1]. Although HKGs are expressed by any cell, their expression varies among different cell types/organs [8,9]. Use of HKGs as reference genes for a particular sample type should be, therefore, validated.

So far, only few reference genes have been validated for cells from respiratory compartment; specifically GNB2L1 was validated for bronchoalveolar macrophages in patients with chronic obstructive pulmonary disease (COPD) [10] and GAPDH (glyceraldehyde-3-phosphate dehydrogenase) for non-small cell lung cancer [11]. The majority of studies published on qRT-PCR in lung setting uses a general approach of normalisation against GAPDH or ACTB (beta-actin) [12-16]. However, these "traditional" reference genes have been already found unsuitable for normalising of mRNA levels in asthmatic airways [17,18] and also for expression studies employing bronchoalveolar macrophages [10].

In order to identify suitable reference genes for qRT-PCR normalisation in the setting of bronchoalveolar compartment, our aim, therefore, was to identify HKGs with the most stable mRNA expression in bronchoalveolar (BAL) cells. Our choice of candidate HKGs was based on 1) their common use in previous qRT-PCR experiments (ACTB, GAPDH, G6PD), 2) stable expression in different human tissues in microarray experiments (ARF1, CANX, GPS1, PSMB2, PSMD2) [8,9], and 3) stable expression in bronchoalveolar macrophages and peripheral neutrophils (GNB2L1, RPL32) [10,19]. To account for variations of BAL cellular profile in different respiratory diseases, we studied stability of HKGs mRNA expression in seventy-one subjects across a spectrum of lung pathologies. Besides BAL cellular profile and type of lung pathology, four variables were investigated for their possible influence on mRNA expression of studied HKGs; these were: smoking, gender, treatment, and age. Further, mRNA expression stability of all ten HKGs was validated in the second, independent BAL cohort consisting of seventeen control subjects and sixty-three sarcoidosis patients with special emphasis on patient subgroups. Finally, by investigation of mRNA expression of two cytokines known associated with sarcoidosis, INFG (interferon gamma) and CCL2/MCP-1, we provided practical evidence, that normalisation with validated reference genes in clinical samples is absolute prerequisite for obtaining clinically unbiased valid information from qRT-PCR.

## Methods

### Subjects

BAL was performed according a standard procedure [20] in 71 Caucasian subjects (1st cohort) with lung diseases diagnosed between 2004 and 2006 in one referral centre in the Czech Republic (Faculty Hospital Olomouc). The diagnoses were in compliance with the criteria from the International Statements/Standards of these diseases: 26 patients with interstitial lung diseases (sarcoidosis, idiopathic interstitial pneumonia, secondary fibrosis, asbestosis, lipoproteinosis and silicosis), 19 cancer patients and 26 COPD patients. For clinical and laboratory characteristics of studied subjects see Table E1 in the Additional file 2.

The subgroups based on gender (45 males/26 females), smoking status (28 smokers/40 non-smokers), treatment before BAL (24 untreated/47 treated), age (median age of 60 years as the division point; 36 patients >60 years/35 patients ≤ 60 years), and groups with normal (N)/pathological (P) differential BAL cell counts were also analyzed. The reference values for BAL cell counts (≥ 85% macrophages, ≤ 11% lymphocytes, <3% neutrophils, ≤ 1% eosinophils) were based on our own laboratory values and correspond to Meyer [21]. The subgroups according BAL cell composition were as follows: 37 N/28 P macrophage-, 46 N/19 P lymphocyte-, 48 N/17 P neutrophil- and 45 N/20 P eosinophil-counts.

The second cohort, used for validation of mRNA expression stability of studied HKGs, consisted of 80 subjects: 63 patients with pulmonary sarcoidosis and 17 control subjects. The control group consisted of subjects (11 males, 6 females; 11 non-smokers, 5 smokers, 1 subject with unknown smoking history; age 42.2 ± 15.7 yrs) undergoing BAL within medical examination for "non-inflammatory condition" e.g. psychogenic cough. All had normal BAL fluid cytology, immunology, and microbiology & CD4+/CD8+ ratio. For clinical and laboratory characteristics of studied subjects from the second cohort see Table E1 in the Additional file 2. None of the patients in the second cohort received corticosteroid therapy before BAL. The subgroups in the second cohort were based on gender (37 males/43 females), smoking status (23 smokers/56 non-smokers), and presence of lung disease (63 sarcoidosis patients/17 control subjects). Further subgroups were formed within the sarcoidosis patient group: based on the presence/absence of Löfgren's syndrome (LS) (11 patients with LS/52 patients without LS), involvement of parenchyma (17 patients with chest X-ray stage I/46 patients with chest X-ray stages II and III), involvement of other organs than lung (40 patients with only involvement of lung/23 patients with multiorgan involvement) and groups with normal (N)/pathological (P) differential BAL cell counts. The subgroups according BAL cell composition were as follows: 15 N/48 P macrophage-, 14 N/49 P lymphocyte-, 56 N/7 P neutrophil- and 58 N/5 P eosinophil-counts.

The study was approved by the Ethics Committee of the Medical Faculty Palacky University & Faculty Hospital Olomouc. All subjects signed informed consent about usage of an aliquot of BAL sample, taken primarily for diagnostic purposes, also for the research purposes of this study.

### BAL sample processing

BAL cells (0.5–1.5 × 10^6^) were separated from the BAL fluid and washed as previously described [22]. Briefly, BAL samples were filtered through one gauze layer followed by separation of BAL cells by centrifugation (400 g, 4°C). The cells were washed twice with 10 ml ice-cold PBS-DEPC, counted and resolved in 50 μl PBS-DEPC. After immediate addition of RNAlater (300 μl; Ambion, Austin, TX, USA), the cells were stored at 4°C overnight and then at -20°C until use. The time between BAL procedure and processing of sample did not exceeded 2 hours.

### Total RNA isolation and quality assessment, reverse transcription

The cells stored in RNAlater were recovered by centrifugation (4000 g, 4°C, 45 min) after 1:2 dilution with ice-cold PBS-DEPC as recommended by the manufacturer. Total RNA was isolated using mirVana miRNA kit (Ambion) and genomic DNA was eliminated by TurboDNAfree kit (Ambion) according to the manufacturer's recommendation. The quantity and quality of RNA samples were assessed by 2100 Bioanalyzer (Agilent Technologies, Palo Alto, USA) using RNA 6000 Nano assays.

Reverse transcription (0.5 μg total RNA, total volume of 20 μl) was performed with Reverse-iT RTase Blend using anchored dT primers (0.4 μg; ABgene, Epsum, U.K.) at 47°C for 45 min in triplicates and then combined. All cDNA samples were diluted to 4 ng input total RNA/μl and stored in aliquots at -20°C until use.

### Gene expression measurements by qRT-PCR

Fluorescently labelled Locked Nucleic Acid probes (LNA, Universal ProbeLibrary; Roche Applied Science, Indianapolis, USA) and the primers (Metabion, Munich, Germany) for investigated genes (Table 1) were selected using ProbeFinder assay design tool  (Table 2). PCR reaction mixes were prepared as follows: equal amount of cDNA (5 μl, corresponding to 20 ng calculated on input total RNA) for each individual gene was added to 20 μl PCR-Mix (ABgene). The final concentrations were 900 nM each sense and antisense primers, 100 nM LNA probe, 3.5 mM MgCl2, 200 μM each dNTPs, 1 U Thermo-Start TAQ polymerase, 1× Thermo-Start Buffer (ABgene). After initial denaturation (one cycle at 94°C for 15 min), 40 cycles amplification (94°C for 45 s, 60°C for 30 s) were performed on RotorGene 3000 system (Corbett Research, Sydney, Australia).

**Table 1:** Description of investigated genes.

**Gene abbreviation**	**Gene name (synonyms)**	GenBank* Accession number	**Function**
ACTB	actin, beta	NM_001101.2	Cytoskeletal structural protein
ARF1	ADP-ribosylation factor 1	NM_001658.3	Activator of phospholipase D
CANX	calnexin	NM_001746.3	Molecular chaperone
G6PD	glucose-6-phosphate dehydrogenase	X03674.1	NADPH production
GAPDH	glyceraldehyde-3-phosphate dehydrogenase	NM_002046.3	Glycolysis enzyme
GNB2L1	Homo sapiens guanine nucleotide binding protein (G protein), beta polypeptide 2-like 1	NM_006098.4	Receptor for activated C-kinase
GPS1	G protein pathway suppressor 1	U20285.2	G protein suppressor
PSMB2	proteasome (prosome, macropain) subunit, beta type, 2	NM_002794.3	Peptide cleavage
PSMD2	26S proteasome subunit p97	D78151.1	Peptide cleavage
RPL32	ribosomal protein L32, transcript variant 1	NM_000994.3	Member of 80 different ribosome proteins
INFG	interferon gamma	NM_000619.2	Cytokine
CCL2	CC chemokine ligand-2/MCP-1	NM_002982.3	Chemotactic cytokine

*Gene sequences available online at

**Table 2:** Characteristics of used primers; LNA probes and amplicon sizes in reverse transcriptase-polymerase chain reaction reaction.

**Gene abbreviation**	**Amplicon size (basepairs)**	**Sense, antisense primers**	**LNA probe¥**
ACTB	76	5'-attggcaatgagcggttc-3' 5'-ggatgccacaggactccat-3'	#11

ARF1	70	5'-gccactacttccagaacacaca-3' 5'-tcgttcacacgctctctgtc-3'	#56

CANX	108	5'-aacaccagaactcaacctgga-3' 5'-tgtcggaagatgaagtgcag-3'	#55

G6PD	75	5'-ctggtggccatggagaag-3' 5'-gcatttcaacaccttgacctt-3'	#22

GAPDH	78	5'-tccactggcgtcttcacc-3' 5'-ggcagagatgatgaccctttt-3'	#45

GNB2L1	72	5'-gctactaccccgcagttcc-3' 5'-cagtttccacatgatgatggtc-3'	#55

GPS1	66	5'-gcaaccagatccatgtcaagt-3' 5'-tgttggctggagtcagctc-3'	#36

PSMB2	72	5'-agagggcagtggaactcctt-3' 5'-aggttggcagattcaggatg-3'	#50

PSMD2	68	5'-gcctcacccagattgacaag-3' 5'-ggcaagaagagctcctgactta-3'	#82

RPL32	75	5'-gaagttcctggtccacaacg-3' 5'-gcgatctcggcacagtaag-3'	#17

INFG	112	5'-ggcattttgaagaattggaaag-3' 5'-tttggatgctctggtcatctt-3'	#21

CCL2	93	5'-agtctctgccgcccttct-3' 5'-gtgactggggcattgattg-3'	#40

¥Numbers of LNA probes according to the commercially available library

Relative expression was calculated using second derivative method (Additional file 3) (RotorGene Software 6.1.71, Corbett Research) as follows: Expression = average amplification^(*CTtcalibrator*-*CTtsample*)^. cDNA from human universal reference RNA (Stratagene, La Jolla, CA, USA) was used as calibrator (in quadruplicates) at concentration of 1.25 ng/reaction calculated on input RNA. For definition of the second derivative method, Takeoff point (CTt) and average amplification see Additional file 3.

### Statistical analysis

Descriptive statistics, F-test for CTt variance equality, Kolmogorov-Smirnov test for normality of log-transformed relative expression values were calculated by software SPSS 13.0 (SPSS Inc, Chicago, IL, USA). Log-transformed relative expression values for INFG and CCL2 were used for statistical calculations by Student's t-test, one-way ANOVA. *P *< 0.05 was considered significant. Equivalence test [23,24], statistical applets BestKeeper [25], geNorm [26] and NormFinder [27] were used for the analysis of gene expression stability. Normalisation factors (NF) for genes and gene pairs were calculated according to Vandesompele et al [26]. For more details on statistical approaches and calculation of normalisation factor see the Additional file 1.

## Results

### Quality of RNA isolated from BAL samples

All investigated RNA samples were of good quality, mean RIN (RNA Integrity Number) values (± S.D.) were 7.4 ± 1.0 (range from 5.5 to 8.6). Among all samples, ratios 28S:18S varied between 1.0–1.4 with no visible degradation products (Fig. E1 in Additional file 4).

### Amplification efficiency and reproducibility of qRT-PCR with fluorescently labelled LNA-probes

In order to determine the amplification efficiency for all studied genes, 5-point standard curves with known concentrations of transcribed human universal reference RNA were constructed. The amplification efficiencies of LNA-based qRT-PCR for studied HKGs varied between 95 to 100%, except for ARF1 where the amplification efficiency of 85% was achieved. The linear regression coefficient (R^2^) for all ten genes ranged between 0.998–0.999. Based on 16 replicates, intra-assay variation of less than 0.7% and inter-assay variation of less than 1.6% were achieved. Negative controls using not transcribed RNA samples for all genes were negative.

### Gene expression levels of ten housekeeping genes within the whole 1st cohort sample set

**Figure 1:**
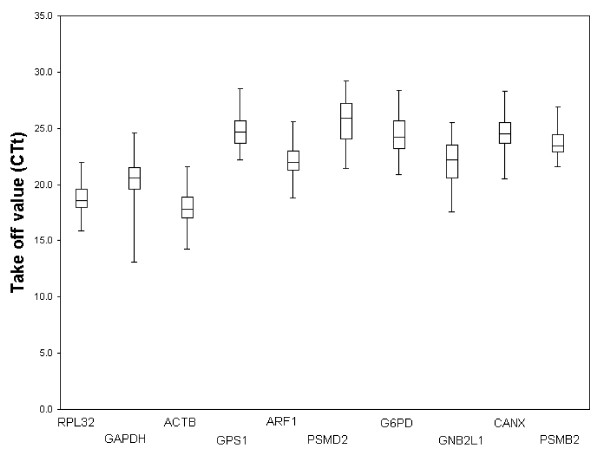
**Expression levels of ten housekeeping genes in bronchoalveolar cells from the 1st cohort**. Expression levels of ten HKGs in CTt values over all BAL samples (n = 71). The data are expressed as whisker box plots; the box represents the 25th–75th percentiles, the median is indicated by a bar across the box, the whiskers on each box represent the minimum and maximum values.

In order to evaluate gene expression levels of all studied HKGs within the whole patient sample set of the 1st cohort, mRNA expressions for every gene were measured in individual BAL samples. Gene expression levels in individual samples showed a broad range of variance between CTt 13.1 (for GAPDH) and CTt 29.20 (for PSMD2) (Fig. 1). Out of ten studied genes, ACTB (mean CTt 17.92) and RPL32 (mean CTt 18.65) were expressed at the highest levels; PSMD2 (mean CTt 25.55) and GPS1 (mean CTt 24.86) at the lowest levels in BAL cells. The lowest expression variability within all samples was observed for the gene PSMB2 (mean CTt ± SD, 23.66 ± 0.86) and RPL32 (18.65 ± 0.92). Genes PSMD2 (25.55 ± 1.67) and GNB2L1 (21.97 ± 1.54) showed the most variable expression within the sample set. F-test showed that PSMB2 and RPL32 had significantly lower variance of CTt values when compared to CANX, GNB2L1, ACTB, PSMD2, ARF1, GPS1, G6PD and GAPDH (*p *< 0.02). Descriptive statistics of gene expression data and corresponding absolute x-fold change values for all studied genes calculated by the applet Bestkeeper are shown in Table 3.

**Table 3:** Descriptive and correlation analysis for ten housekeeping genes in the 1st cohort obtained by BestKeeper statistical applet.

	RPL32*	**GAPDH**	**ACTB**	**GPS1**	**ARF1**
GM [CTt]	18.65	20.44	17.92	24.86	22.15
AM [CTt]	18.69	20.53	17.99	24.91	22.20
Min [CTt]	15.90	13.10	14.30	22.20	18.80
Max [CTt]	22.00	24.60	21.60	28.50	25.60
SD [± CTt]	0.92	1.34	1.21	1.24	1.14
CV [% CTt]	4.93	6.51	6.73	4.99	5.12
Min [x-fold]	-6.73	-161.54	-12.31	-6.34	-10.21
Max [x-fold]	10.19	17.93	12.80	12.43	10.92
SD [± x-fold]	1.89	2.53	2.31	2.37	2.20

	**PSMD2**	**G6PD**	**GNB2L1**	**CANX**	PSMB2*

GM [CTt]	25.55	24.36	21.97	24.42	18.65
AM [CTt]	25.63	24.42	22.05	24.48	18.69
Min [CTt]	21.40	20.90	17.60	20.50	15.90
Max [CTt]	29.20	28.40	25.50	28.30	22.00
SD [± CTt]	1.67	1.34	1.54	1.25	0.92
CV [% CTt]	6.51	5.48	7.00	5.10	4.93
Min [x-fold]	-17.81	-11.03	-20.63	-15.17	-6.73
Max [x-fold]	12.51	16.42	11.58	14.69	10.19
SD [± x-fold]	3.18	2.53	2.91	2.38	1.89

Definition of abbreviations: GM [CTt], geometric mean of CTt; AM [CTt], arithmetic mean of CTt; Min [CTt] and Max [CTt], minimum and maximum values of CTt; SD [± CTt], standard deviation of the CTt; CV [%CTt], coefficient of variance expressed as a percentage on the CTt level. Min [x-fold] and Max [x-fold]: the extreme values of expression levels expressed as an absolute x-fold over- or under- regulation coefficient; SD [± x-fold]: standard deviation of the absolute regulation coefficients.

* Stably expressed genes were selected according to the criteria (SD [± CTt] < 1) published in Pfaffl et al [25].

### Analysis of expression stability of ten HKGs in BAL cells from the 1st patient cohort by equivalence test and statistical applets Bestkeeper, geNorm and NormFinder

In order to find out the most suitable reference genes for normalisation of gene expression in BAL cells, four different statistical approaches (equivalence test, applets Bestkeeper, geNorm and NormFinder) were applied in parallel to assess the gene expression stability of ten HKGs within the whole sample set and also in patient subgroups based on gender, smoking status, treatment, disease type, age, and BAL differential cell counts.

#### a) Equivalence test

**Figure 2:**
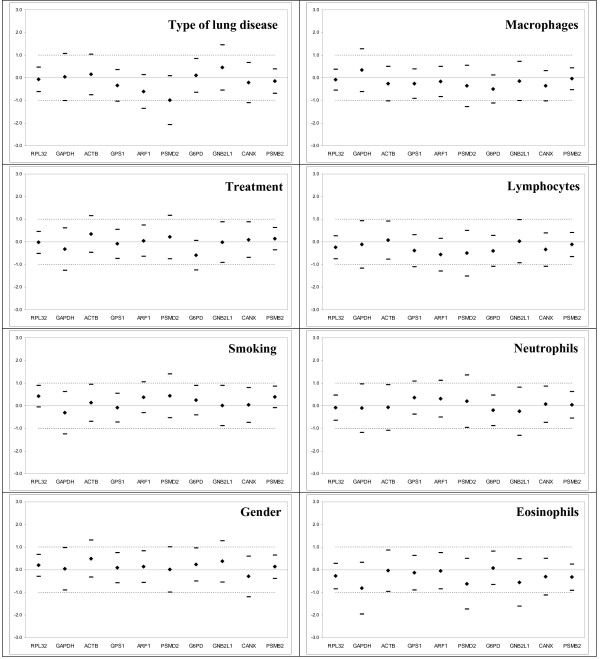
**Equivalence test for ten housekeeping genes in the 1st cohort subgroups based on the type of lung disease, treatment, smoking status, gender, and bronchoalveolar lavage cellular composition**. Differences of the means (◆) and matching symmetrical confidence intervals (-) are shown for the log2-transformed relative expression of HKGs. Y-axis represents the fold change in expression among subgroups. The deviation area [-1; 1] for a fold change ≤ 2 lies within the dashed lines. If the symmetrical confidence interval is a part of the deviation area and contains zero in them, the gene is considered to be expressed equivalently. For more details on calculation see the Additional files and for statistical methodology the references [23,24]. Mean differences were calculated as follows: Mean(interstitial diseases)-Mean(other lung diseases), Mean(treated)-Mean(untreated), Mean(males)-Mean(females), Mean(smokers)-Mean(non-smokers), and Mean(pathological BAL cell counts)-Mean(normal BAL cell counts) for macrophages, lymphocytes, neutrophils and eosinophils. Reference BAL cell counts were based on own laboratory values and correspond to Meyer [21], for more details see Methods section.

**Figure 3:**
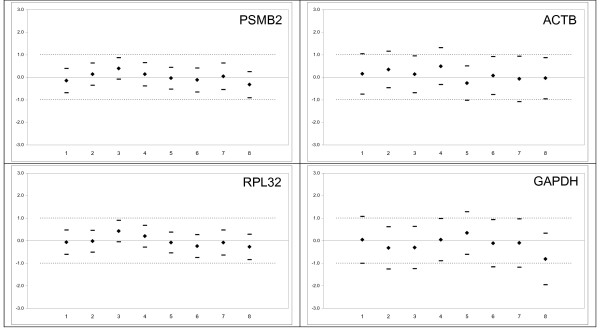
**Comparison of results of equivalence tests in all studied subgroups of the 1st cohort for two most stable genes PSMB2 (the left upper part) and RPL32 (the left lower part) and two most commonly used genes ACTB (the right upper part) and GAPDH (the right lower part) in bronchoalveolar (BAL) cells**. 1 – Type of lung disease; 2 – Treatment; 3 – Smoking status; 4 – Gender; 5 – BAL Macrophage count; 6 – BAL Lymphocyte count; 7 – BAL Neutrophil count; 8 – BAL Eosinophil count. For more details see the legend to Fig. 2.

In order to identify the most stably expressed genes in patient subgroups by equivalence test, we applied two-fold expression change cut-off for group-wise comparisons. Genes GAPDH and PSMD2 were identified as the least stably expressed genes in BAL samples, equivalently expressed only in subgroups according gender and age (Fig. 2, data for age comparison not shown). Genes ARF1, ACTB, CANX, GAPDH, GNB2L1, G6PD, GPS1, PSMD2 were found not equivalently expressed in more than two of eight studied subgroups. Out of all studied genes, only PSMB2 and RPL32 were found equivalently expressed in all studied subgroups (Fig. 2). The comparison of results of equivalence tests for two most stable genes (PSMB2, RPL32) and two "traditional" reference genes (ACTB, GAPDH) in all subgroups is shown in Fig. 3.

#### b) Analysis by BestKeeper

Analysis by the applet BestKeeper showed that only two genes (PSMB2 and RPL32) are stably expressed within the whole data set (Table 3), as well as in all studied subgroups based on gender, smoking status, treatment, type of the disease, age and BAL cellular composition (data not shown). The expression of genes ACTB, ARF1, CANX, GAPDH, G6PD, GPS1, GNB2L1 and PSMD2 has to be considered as inconsistent (standard deviation of the CTt value > 1), thus they were excluded from further analysis. When the regression analysis was performed with two stable genes, the PSMB2 was shown to be more suitable as a reference gene (coefficient of correlation, r = 0.914; *p *= 0.001) than RPL32 (r = 0.865; *p *= 0.001).

#### c) Analysis by geNorm

Average expression stability measure of ten HKGs in the whole sample group during stepwise exclusion of the least stable genes by the applet geNorm resulted in following gene order: the most stable-ACTB-GPS1-GNB2L1-ARF1-PSMB2-RPL32-CANX-G6PD-PSMD2-GAPDH-the least stable. When the applet was applied to particular subgroups, we obtained various ranking lists of suitable reference genes for various subgroups: e.g. ACTB and GNB2L1 were the most stable genes for smokers and ACTB and PSMB2 for non-smokers; PSMB2 and RPL32 were the most stable genes for males and ACTB and GNB2L1 for females.

#### d) Analysis by NormFinder

Analysis by the applet NormFinder ranked ten genes according their expression stability in the whole patient set in the following order: the most stable-ACTB-PSMB2-GNB2L1-ARF1-GPS1-RPL32-CANX-G6PD PSMD2-GAPDH-the least stable. However, we obtained various ranking lists when we calculated the expression stability of ten investigated HKGs in subgroups: e.g. genes PSMB2 and ARF1 were the most stable genes in subgroups based on smoking status (smokers vs. non-smokers), ACTB and GNB2L1 were the most stable genes in subgroups based on gender (males vs. females).

### Validation of expression stability of ten HKGs in BAL cells from the 2nd patient cohort by equivalence test

**Figure 4:**
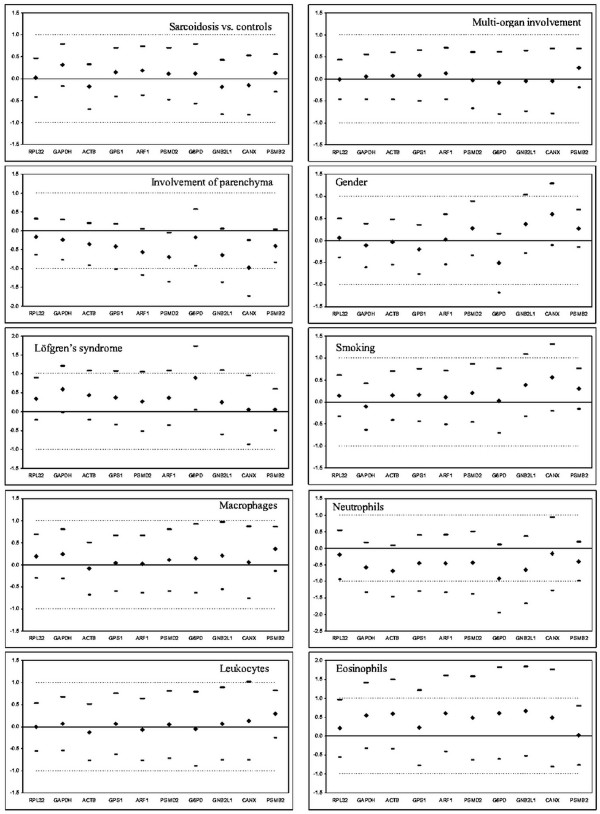
**Equivalence test for ten housekeeping genes in the 2nd cohort subgroups based on the presence of disease, involvement of parenchyma, presence of Löfgren's syndrome, multi-organ involvement, smoking status, gender, and bronchoalveolar lavage cellular composition**. Mean differences were calculated as follows: Mean(sarcoidosis patients)-Mean(control subjects), Mean(patients with involvement of parenchyma: CXR stages II/III)-Mean(patients without involvement of parenchyma: CXR stage I), Mean(Löfgren's syndrome patients)-Mean(non-Löfgren's syndrome patients), Mean(multi-organ involvement)-Mean(involvement of lung only), Mean(smokers)-Mean(non-smokers), Mean(males)-Mean(females), and Mean(pathological BAL cell count)-Mean(normal BAL cell count) for macrophages, lymphocytes, neutrophils and eosinophils. For more details see the legend to Fig. 2.

In order to confirm that PSMB2 and RPL32 genes, identified as the most stable genes in the aforementioned analyses in the 1st cohort, has indeed the most stable mRNA expression unaffected by range of tested variables, we investigated gene expression of all ten genes in the second, independent BAL cohort (63 patients with pulmonary sarcoidosis and 17 control subjects) by equivalence test. The relative gene expression values for all genes were compared among the patient subgroups based on gender, smoking status, and clinical characteristics such as presence of disease, presence of Löfgren's syndrome, involvement of parenchyma, involvement of other organs than lung and BAL differential cell counts (Fig. 4, Fig. E2 in Additional file 5). Out of ten studied genes, only PSMB2 and RPL32 genes were equivalently expressed in all tested subgroups of the second cohort.

### Assessment on the minimal number of reference genes for normalisation of qRT-PCR in BAL cells

In order to evaluate the minimal number of reference genes for normalisation of qRT-PCR in BAL cells, we calculated the normalisation factors for novel reference genes and their combination (PSMB2, RPL32, PSMB2-RPL32) and for the "traditional" reference genes in lung settings (ACTB, GAPDH) in all individual samples in both cohorts separately. Generally, the most suitable reference genes are the genes with mean NF value closest to 1 and with the lowest SD. Gene PSMB2 alone showed the lowest mean NF, SD and coefficient of variation (CV) in both cohorts (1. cohort: mean NF ± SD, CV: 1.27 ± 0.89, 70%; 2. cohort: 1.17 ± 0.63, 54%). Gene RPL32 alone showed in both cohorts the same mean NF as PSMB2 gene, but higher SD and CV (1. cohort: 1.27 ± 0.93, 73%; 2. cohort: 1.17 ± 0.71, 61%). Pairing of PSMB2 with RPL32 did not significantly improve the mean NF value and the variability (1. cohort: 1.29 ± 0.91, 72%; 2. cohort: 1.14 ± 0.59, 52%) compared to PSMB2 or RPL32 alone. Genes ACTB (1. cohort: 1.64 ± 1.52, 93%; 2. cohort: 1.20 ± 0.75, 63%) and GAPDH (1. cohort: 1.60 ± 2.98, 186%; 2. cohort: 1.22 ± 0.76, 62%) were found less suitable as reference genes for BAL cells. We, therefore, recommend single genes PSMB2 and RPL32 as denominators for gene expression studies in BAL cells.

### Effect of the used reference gene on relative target gene expression values: study of mRNA expression of INFG and CCL2 known to be associated with pulmonary sarcoidosis (2. cohort)

In order to demonstrate the effect of used reference genes on the result of target gene expression data in BAL cells, we investigated relative mRNA expression of two cytokines known to be associated with sarcoidosis, INFG and CCL2, in sarcoidosis patients and control subjects (2nd cohort). The following genes were applied as denominators: 1) reference genes validated in our study (PSMB2, RPL32) and 2) "traditional" reference genes (ACTB, GAPDH). The data are presented as a mean fold change of relative expression compared to control subjects (normalized to 1).

**Figure 5:**
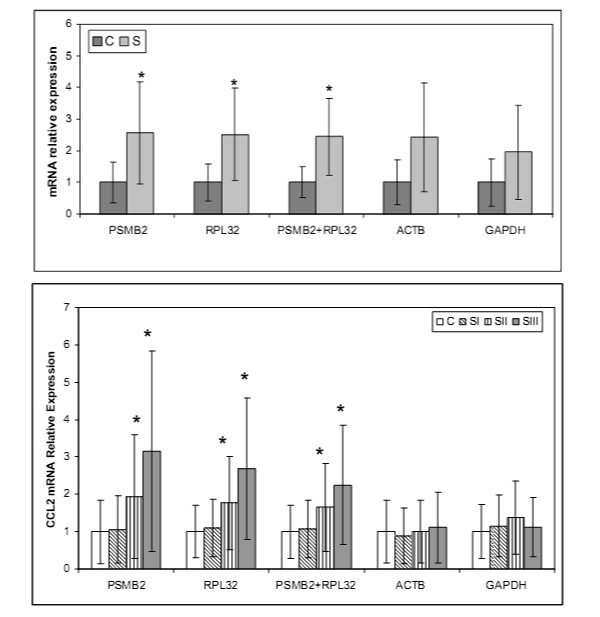
**Comparison between the relative mRNA expression (ratio target gene/reference gene) of INFG gene (the upper part) and CCL2 gene (the lower part) in unseparated bronchoalveolar cells of sarcoidosis patients (S, n = 63) and control subjects (C, n = 17) from the 2nd cohort using newly validated (PSMB2/RPL32) and "traditional" reference genes (ACTB/GAPDH) as denominators**. The data are presented as a mean fold change of relative expression compared to control subjects (normalized to 1); the whiskers on each box represent the SD values. For details see Methods section Gene expression measurements by qRT-PCR. *p < 0.05.

Relative mRNA expression levels of INFG were higher in sarcoidosis patients than in control subjects when the normalisation was done with gene PSMB2 (fold change ± SD: 2.56 ± 1.62; *p *= 0.004), with gene RPL32 (2.58 ± 1.46; *p *= 0.004), and with gene pair PSMB2-RPL32 (2.44 ± 1.20; *p *= 0.02) (Fig. 5). When the expression level of INFG was normalised to ACTB (2.42 ± 1.71; *p *= 0.053) or to GAPDH (1.95 ± 1.48; *p *= 0.09), the mRNA expression of INFG did not differ between control subjects and sarcoidosis patients (Fig. 5).

Similar, when CCL2 mRNA levels were expressed as a ratio to ACTB (1.00 ± 0.85; *p *= 0.46) or to GAPDH (1.37 ± 0.98; *p *= 0.43), there were not significant differences in mRNA levels in BAL cells between sarcoidosis patients with chest radiographic stage 2 and stage 1 patients. Using genes PSMB2 (1.95 ± 1.66; *p *= 0.02) and gene RPL32 (1.77 ± 1.25; *p *= 0.03), and gene pair PSMB2-RPL32 (1.65 ± 1.17; *p *= 0.02) as denominators, CCL2 mRNA levels differed between chest radiographic stage 2 and stage 1 sarcoidosis patients (Fig. 5).

## Discussion

Aiming at finding suitable reference genes for quantitative gene expression profiling studies in bronchoalveolar cells, we have investigated the gene expression of ten housekeeping genes selected according their expression stability reported in literature or their common use in qRT-PCR. Out of these, two genes PSMB2 and RPL32 were found constantly expressed in unseparated BAL cells from seventy-one subjects irrespective of lung pathology, smoking status, gender, treatment, age and BAL cellular composition. The stability of mRNA expression of PSMB2 and RPL32 genes was further validated in the second, independent BAL cohort of sixty-three sarcoidosis patients and seventeen control subjects. By contrast to PSMB2 and RPL32, expression levels of genes ACTB, ARF1, CANX, GAPDH, G6PD, GPS1, GNB2L1 and PSMD2 considerably varied among studied patient subgroups in both investigated cohorts thus making these genes less suitable for the normalisation in qRT-PCR. We, therefore, recommend PSMB2 and RPL32 as suitable reference genes for the normalisation of the gene expression in unseparated BAL cells, namely in interstitial lung diseases. Moreover, based on our data, PSMB2 and RPL32 represent promising candidate reference genes for other lung pathologies such as COPD and cancer. Finally, we demonstrated on the example of INFG and CCL2 mRNA expression in sarcoidosis that the normalisation with validated reference genes in clinical samples is absolute prerequisite for obtaining clinically meaningful information from qRT-PCR.

Although qRT-PCR is an established method for quantifying of mRNA expression in BAL samples, normalisation for differences among individual samples is the major difficulty of this methodology [1,7]. Several normalisation strategies can be applied: normalisation to sample volume, to total RNA and to internal reference genes or their combination. Normalisation to equal volumes on its own is not suitable for respiratory settings because BAL samples differ in cell counts and cellular composition. The other approach, the normalisation for quantity of total RNA, is disqualified because it does not correct for differences in RNA quality and in reverse transcriptase efficiencies among samples [28]. Nowadays, the endogenous reference genes represent the most suitable and easiest way for normalisation of clinical samples in qRT-PCR [1,29-32]. Moreover, reference genes may correct also for differences in RNA integrity among the samples [33,34]. Similarly to Huggett et al [3], we affirm that the combination of similar sample size, similar RNA concentration in reverse transcription and use of validated reference genes represents the proper normalisation strategy for BAL samples.

Although it is known that the normalisation with unsuitable reference gene may lead to misinterpretation of target genes expression data [17,35], most investigators have generally used the genes GAPDH and ACTB as reference genes to normalize qRT-PCR in lung settings without previous validation [12-16]. The reason may be the fact that the known approaches for validation of reference gene stability have been introduced mainly for cell cultures and tissues [2,26,27,36] and no general approach for validation of reference genes in clinical samples is recommended nowadays. In our study, we applied four different mathematical and statistical models to select stably expressed HKGs genes in BAL samples. Similarly to Robinson et al [37], we observed that the output of the most suitable reference genes using pair-wise approach geNorm [26] is influenced by chosen set of candidate genes, and the ranking of the genes occurs according the similarity in expression profiles [27]. Another applet, the model-based approach NormFinder [27], takes already into account the individual gene expression variability and calculates the gene expression stability in subgroups. By contrast to Andersen et al [27], who compared gene expression in two types of cancer tissues, we aimed to investigate the influence of many variables (e.g. gender, smoking, age, BAL cellular composition, lung pathology and treatment) on the expression stability of studied genes. Doing so, we obtained various ranking lists of suitable reference genes for various subgroups, thus making NormFinder approach less suitable for our purpose. Using the third used approach, the BestKeeper applet, only PSMB2 and RPL32 were revealed as stably expressed genes in BAL samples, the eight remaining genes were excluded from further analyses as inconsistently expressed [25]. Moreover, the limitation of this approach is the use of Pearson's correlation [25], which makes it unsuitable for analyses of non-normally distributed data commonly observed in clinical sample sets. The heterogeneity of the results obtained by the statistical applet Bestkeeper, geNorm and NormFinder and also having in mind our aim to identify genes stably expressed irrespective of many variables (gender, smoking, BAL cellular composition, lung pathology and medication) contributed to our final decision to apply equivalence test into our analyses of HKGs expression stability in BAL cells. Doing so, to exclude genes with high expression variability within the sample set and studied subgroups, we set very strict criteria corresponding to two-fold change in gene expression [24]. Similarly to previous reports [10,17] we observed that "traditional" reference genes like ACTB and GAPDH are indeed unsuitable for normalisation of gene expression in BAL cells. Even in the case of GNB2L1, gene recommended as a reference gene for BAL macrophages from COPD patients [10], we observed that its expression in BAL cells is influenced by lung pathology, treatment and by eosinophil and neutrophil counts in BAL samples. Only two genes, PSMB2 and RPL32, were found constantly expressed in all studied subgroups irrespective of smoking, gender, treatment, age, lung pathology and BAL cellular composition. To enhance the evidence about invariable expression of PSMB2 and RPL32 genes, their expression was further validated in second, independent BAL cohort of patients with sarcoidosis and control subjects. We are aware that we dealt mostly with bronchoalveolar cells from interstitial lung diseases and sample sizes of other diseases have been limited. Addressing this limitation in the future is prerequisite for definite conclusion about general usage of PSMB2 and RPL32 as reference genes for expression studies in lung compartment as a whole.

PSMB2 belongs to the group of genes encoding for constitutively expressed 20S proteasomal core subunits, RPL32 is a gene encoding for a component of the 60S ribosomal subunit. Various ribosomal proteins have been already validated for qRT-PCR: RPL13A for the pancreas and the prostate tissues [38], LRP10 for adipose tissue [39], RPL32 for human neutrophils [19] and BAL macrophages from COPD patients, where it was stable irrespective of disease severity [10]. PSMB2 showed only 29% variation in expression among 19 human tissues by microarray technique [8] and here we show for the first time its suitability as a reference gene for qRT-PCR also in unsepared BAL cells.

There has been ongoing discussion about the minimal number of reference genes required for qRT-PCR in clinical samples. Although the combination of more than one normalisation gene resulted in improved accuracy in several studies [26,27,40-42], other investigators showed that normalisation with a single gene is sufficient for most research applications [36,43-45]. Also our analyses showed that the combination of two most stable genes (PSMB2 and RPL32) did not yield improved precision over normalisation with PSMB2 or RPL32 genes alone. We, therefore, suggest that the use of single reference genes PSMB2 or RPL32 is sufficient for normalisation of target gene expression in BAL cells, at least in interstitial lung diseases, where we validated their expression stability in the second, independent BAL cohort. PSMB2 gene is a moderate-copy gene, thus can better control for RNA isolation efficiency, RNA quality and RT-efficiency than RPL32 expressed at high abundance.

In order to demonstrate that the normalisation with reference genes with variable expression may indeed lead to the misinterpretation of target gene expression and even to missing the identification of clinically relevant molecules, we applied the newly defined reference genes for investigation of mRNA levels of two cytokine genes reported to be associated with sarcoidosis. These were: Th1 cytokine INFG, which mRNA and protein was elevated in Th1 polarised sarcoidosis [46,47] and CC chemokine ligand (CCL)-2/MCP-1, implicated in the development of sarcoid alveolitis namely in chest X-ray stage 2 disease [22]. In our patients, increase of INFG mRNA in sarcoid BAL cells was observed only when PSMB2/RPL32 were used as denominators in the normalization procedure. Controversially, normalization of INFG transcripts to ACTB/GAPDH did not resulted in INFG mRNA up-regulation. Similarly, CCL2 mRNA up-regulation in sarcoid chest X-ray stage 2 disease was observed when stably expressed reference genes PSMB2/RPL32 were used. Use of ACTB/GAPDH as denominators again yielded inconclusive, ambiguous expression data. By these reports we emphasize that our results provide an important and clear message for pulmonary science because only using validated (i.e. stably expressed) reference genes for normalization will one ensure that detected changes in target gene expressions in BAL samples are valid and therefore clinically meaningful. By contrast, usage of genes with variable expression such as ACTB or GAPDH for normalization leads to misinterpretation of target gene expression in lung samples.

## Conclusion

In conclusion, our study aimed at identifying stable genes, the expression of which is not influenced by variables such as smoking, gender, age, lung pathology, treatment and BAL cellular composition. Genes PSMB2 and RPL32 fulfilled the above criteria, and, therefore, they represent suitable normalisation genes for qRT-PCR in bronchoalveolar cells, namely for studies in sarcoidosis and other interstitial lung diseases.

## Authors' contributions

EK as the main author conceived, designed and interpreted the study and was the primary author of the drafts and of the final version of the paper. AA performed the statistical analysis and contributed to writing the paper. RF performed the gene expression analyses and collected the clinical and gene expression data. FM and ZN collected the clinical patient characteristics. VK and JZ performed the bronchoalveolar lavage, selected the patients and helped to collect the clinical patient characteristics. RdB helped to design the study and contributed to writing the paper. MP is the person responsible for the integrity of the study; he participated in study conception & design, sample acquisition, interpretation & writing the final version of the paper. All authors read and approved the final manuscript.

## Competing interests

The authors declare that there are no competing interests.

## Supplementary Material

Additional file 1 Description of used statistical approaches. Click here for file

Additional file 2 **Table E1**. Clinical and laboratory characteristics of investigated subjects. Click here for file

Additional file 3 Definition of terms. Click here for file

Additional file 4 **Figure E1. RNA quality assessment (a representative example) by 2100 Bioanalyzer (Agilent Technologies, Palo Alto, USA)**. This figure shows typical chromatogram of microcapillary electrophoresis of total RNA preparation of good quality extracted from bronchoalveolar lavage cells. Electropherogram shows 18S and 28S rRNA peaks. FU – Fluorescence units. Click here for file

Additional file 5 **Figure E2. Expression levels of ten housekeeping genes in bronchoalveolar cells from sarcoidosis patients and normal subjects from the 2nd cohort**. Expression levels of ten housekeeping genes in CTt values in bronchoalveolar cells from sarcoidosis patients (n = 63) a normal subjects (n = 17). The data are presented as means (columns) ± SD (errorbars). White columns represent the control group, dark columns sarcoidosis patients. Click here for file
