# Supplementary material for: PSMB2 and RPL32 are suitable denominators to normalize gene expression profiles in bronchoalveolar cells
Source: BMC Mol Biol. 2008 Jul 31;9:69. doi: 10.1186/1471-2199-9-69 (PMC2529339; doi:10.1186/1471-2199-9-69)
Supplement: Additional file 1 — Description of used statistical approaches. [file 1471-2199-9-69-S1.doc]

**Description of used statistical approaches**

**A) Description of Equivalence test**

Equivalence test [E1] calculates whether the absolute value of true difference is bounded by small positive number ε. This lead to formulation of equivalence hypothesis:

H0 (inequivalence): δ ∉[−ε;ε] vs. H1 (equivalence): δ ∈ [−ε;ε].

Derivation area [−ε;ε] was set to [log_2_(0.5); log_2_(2)] = [-1;1], which corresponds to two-fold change in expression.

A confidence interval CI(δ) = [δL; δH] (where δ is a log_2_ transformed expression values) and calculated as follows:


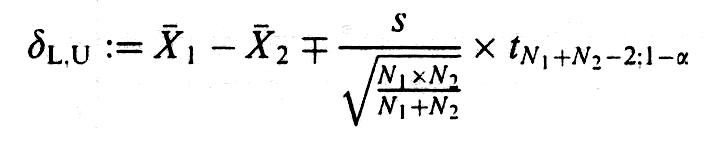


where standard deviation s is calculated by:


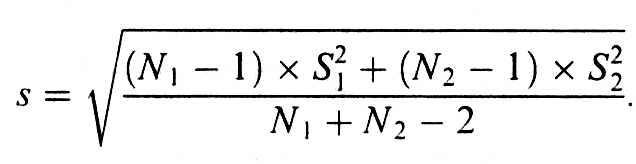


where X_1_ and X_2_ are the mean of log-transformed expression values in groups, N_1_ and N_2_ are the number of samples in groups, S_1_ and S_2_ – standard deviation of log-transformed expression values in groups and t_N1+N2-2_;1-α is a 1-α quartile of the t distribution with N1+N2-2 degrees of freedom.

A gene can be called equivalently expressed if:

Criterion 1: CI(δ) must include 0.

Criterion 2: [δL; δH] ⊂ [−ε;ε].

The constant level α = 0.05 was used for rejection of null hypothesis.

**B) Description of applets BestKeeper, geNorm and NormFinder**

Applet BestKeeper [E2] is an Excel-based tool determining "optimal" reference genes by using a pair-wise correlation analysis (Pearson’s correlation) of all pairs of candidate genes, and calculating geometric mean of the "best" suited ones. Genes exhibiting a standard deviation value of CTt higher than 1 should be considered as inconsistent and excluded from further analysis.

Applet geNorm [E3] calculates the gene expression stability measure (M), which is the mean pair-wise variation for every gene compared with all other tested candidate genes. The most stable genes have the lowest M value; the proposed threshold for eliminating a gene as unstable is M>1.5.

Applet NormFinder [E4] calculates the stability values of the candidate genes based on the combined estimate of intra- and inter-group gene expression variations. Suitable reference genes posses the lowest stability value(s).

**References**

E1. Haller F, Kulle B, Schwager S, Gunawan B, von Heydebreck A, Sultmann H, Fuzesi L: **Equivalence test in quantitative reverse transcription polymerase chain reaction: confirmation of reference genes suitable for normalization.** *Anal Biochem* 2004, **335:**1-9.

E2. Pfaffl MW, Tichopad A, Prgomet C, Neuvians TP: **Determination of stable housekeeping genes, differentially regulated target genes and sample integrity: BestKeeper--Excel-based tool using pair-wise correlations.** *Biotechnol Lett* 2004, **26:**509-15.

E3. [Vandesompele J](http://www.ncbi.nlm.nih.gov/entrez/query.fcgi?db=pubmed&cmd=Search&itool=pubmed_AbstractPlus&term="Vandesompele+J"%5BAuthor%5D), [De Preter K](http://www.ncbi.nlm.nih.gov/entrez/query.fcgi?db=pubmed&cmd=Search&itool=pubmed_AbstractPlus&term="De+Preter+K"%5BAuthor%5D), [Pattyn F](http://www.ncbi.nlm.nih.gov/entrez/query.fcgi?db=pubmed&cmd=Search&itool=pubmed_AbstractPlus&term="Pattyn+F"%5BAuthor%5D), [Poppe B](http://www.ncbi.nlm.nih.gov/entrez/query.fcgi?db=pubmed&cmd=Search&itool=pubmed_AbstractPlus&term="Poppe+B"%5BAuthor%5D), [Van Roy N](http://www.ncbi.nlm.nih.gov/entrez/query.fcgi?db=pubmed&cmd=Search&itool=pubmed_AbstractPlus&term="Van+Roy+N"%5BAuthor%5D), [De Paepe A](http://www.ncbi.nlm.nih.gov/entrez/query.fcgi?db=pubmed&cmd=Search&itool=pubmed_AbstractPlus&term="De+Paepe+A"%5BAuthor%5D), [Speleman F](http://www.ncbi.nlm.nih.gov/entrez/query.fcgi?db=pubmed&cmd=Search&itool=pubmed_AbstractPlus&term="Speleman+F"%5BAuthor%5D): **Accurate normalization of real-time quantitative RT-PCR data by geometric averaging of multiple internal control genes.** [*Genome Biol*](javascript:AL_get(this, 'jour', 'Genome Biol.');) 2002, **3:**RESEARCH0034.

E4. [Andersen CL](http://www.ncbi.nlm.nih.gov/entrez/query.fcgi?db=pubmed&cmd=Search&itool=pubmed_AbstractPlus&term="Andersen+CL"%5BAuthor%5D), [Jensen JL](http://www.ncbi.nlm.nih.gov/entrez/query.fcgi?db=pubmed&cmd=Search&itool=pubmed_AbstractPlus&term="Jensen+JL"%5BAuthor%5D), [Orntoft TF](http://www.ncbi.nlm.nih.gov/entrez/query.fcgi?db=pubmed&cmd=Search&itool=pubmed_AbstractPlus&term="Orntoft+TF"%5BAuthor%5D): **Normalization of real-time quantitative reverse transcription-PCR data: a model-based variance estimation approach to identify genes suited for normalization, applied to bladder and colon cancer data sets.** [*Cancer Res*](javascript:AL_get(this, 'jour', 'Cancer Res.');) 2004, **64:**5245-50.
