# Supplementary material for: PSMB2 and RPL32 are suitable denominators to normalize gene expression profiles in bronchoalveolar cells
Source: BMC Mol Biol. 2008 Jul 31;9:69. doi: 10.1186/1471-2199-9-69 (PMC2529339; doi:10.1186/1471-2199-9-69)
Supplement: Additional file 2 — Table E1. Clinical and laboratory characteristics of investigated subjects. [file 1471-2199-9-69-S2.doc]

**Table E1. Clinical and laboratory characteristics of investigated subjects.**

|  | **1st cohort** | | | | | | **2nd cohort** | | |
| --- | --- | --- | --- | --- | --- | --- | --- | --- | --- |
| **Groups** | **All** | **Interstitial diseases** | | | **Other lung diseases** | |  | **Sarcoidosis and control subjects** | |
|  |  | **Sarcoidosis** | **Idiopathic interstitial pneumonia** | **Other interstitial diseases *** | **Cancer** | **COPD** | **All** | **Sarcoidosis** | **Control subjects** |
| **Number of subjects** | 71 | 8 | 7 | 11 | 19 | 26 | 80 | 63 | 17 |
| **Gender (Female/Male)** | 26/45 | 5/3 | 2/5 | 3/8 | 8/11 | 8/18 | 43/37 | 37/26 | 6/11 |
| **Smoking (Y/N)** | 28/40^✝3^ | 1/6^✝1^ | 1/6 | 4/7 | 7/11^✝1^ | 15/10^✝1^ | 23/56^✝1^ | 18/45 | 5/11^✝1^ |
| **Treatment (Y/N)**** | 47/24 | 5/3 | 6/1 | 6/5 | 15/4 | 15/11 | 0/80 | 0/63 | 0/17 |
| **Age at diagnosis, years** | 59.2±13.8 | 48.0±11.8 | 61.9±12.7 | 52.6±14.6 | 59.3±16.5 | 64.5±9.5 | 46.7±12.4 | 47.9±11.1 | 42.2±15.7 |
|  | (20.0-83.0) | (32.0-62.0) | (46.0-80.0) | (34.0-75.0) | (20.0-81.0) | (44.0-83.0) | (19.0-71.0) | (28.0-71.0) | (19.0-63.0) |
| **BAL differential count** | | | | | | | | | |
| **Macrophage relative count, %** | 82.9±16.8^✝6^ | 76.4±12.9 | 81.1±6.8 | 79.5±14.7^✝1^ | 87.9±9.8 | 82.9±24.5^✝5^ | 78.7±13.9^✝1^ | 75.0±13.1 | 92.9±4.8^✝1^ |
|  | (1.0-99.4) | (59.0-92.0) | (72.0-90.0) | (49.7-98.4) | (66.1-99.1) | (1.0-99.4) | (40.0-99.0) | (40.0-97.1) | (83.8-99.0) |
| **Lymphocyte relative count, %** | 9.3±10.5^✝6^ | 22.1±12.4 | 9.4±7.3 | 12.3±11.9^✝1^ | 7.4±8.6 | 4.8±7.5^✝5^ | 19.0±13.0^✝1^ | 22.4±112.3 | 5.5±4.3^✝1^ |
|  | (0.0-36.5) | (7.0-36.5) | (0.0-19.0) | (0.0-32.3) | (0.0-33.0) | (0.0-32.0) | (0.0-54.0) | (2.0-54.0) | (0.0-13.6) |
| **Neutrophil relative count, %** | 6.4±14.7^✝6^ | 1.0±1.3 | 7.6±2.0 | 7.5±15.0^✝1^ | 2.7±3.9 | 10.9±23.0^✝5^ | 1.8±3.2^✝1^ | 1.9±3.6 | 1.2±1.4^✝1^ |
|  | (0.0-89.0) | (0.0-3.0) | (4.0-9.7) | (0.0-45.0) | (0.3-16.0) | (0.0-89.0) | (0.0-18.0) | (0.0-18.0) | (0.0-3.0) |
| **Eosinophil relative count, %** | 1.4±2.5^✝6^ | 0.6±0.6 | 2.0±1.2 | 0.7±0.9^✝1^ | 2.0±3.7 | 1.3±2.4^✝5^ | 0.6±2.0^✝1^ | 0.7±2.2 | 0.4±0.9^✝1^ |
|  | (0.0-14.0) | (0.0-1.5) | (0.0-3.3) | (0.0-3.0) | (0.0-14.0) | (0.0-10.0) | (0.0-17.0) | (0.0-17.0) | (0.0-1.5) |

Definition of abbreviations: COPD, chronic obstructive pulmonary disease; BAL, bronchoalveolar lavage; N, no; Y, yes.

* Other interstitial diseases: patients with secondary pulmonary fibrosis, lipoproteinosis, asbestosis, silicosis.

** Untreated patients: those, who did not received any therapy at the time of BAL. Treated patients: 15 patients with cancer were on chemotherapy drugs, 17 patients with interstitial diseases and 9 patients with COPD were on corticosteroids, 6 patients with COPD were on corticosteroids in combination with bronchodilators at the time of BAL.

✝ Number indicates for how many patients the data were not available.

Data are presented as means ± SD (minimum and maximum in parentheses).
