# Supplementary material for: PSMB2 and RPL32 are suitable denominators to normalize gene expression profiles in bronchoalveolar cells
Source: BMC Mol Biol. 2008 Jul 31;9:69. doi: 10.1186/1471-2199-9-69 (PMC2529339; doi:10.1186/1471-2199-9-69)
Supplement: Additional file 3 — Definition of terms. [file 1471-2199-9-69-S3.doc]

Definition of terms

| **Term** | **Definition** |
| --- | --- |
| Second derivative method | Quantification method, based on numerical calculation of second derivative of amplification plot that produces peaks corresponding to the maximum rate of fluorescence increase in the reaction. |
| Take Off point (CTt) | Data point on second derivative curve that corresponds to 20 % of the maximum rate of fluorescence, and indicates the end of the noise and the transition into the exponential phase. |
| Amplification efficiency | The efficiency of amplification during PCR. A 100% efficient reaction would result in an amplification value of 2 for every sample, which means that doubling of an amplicon takes place in every cycle.  In the second derivative method amplification efficiency is calculated for each sample by calculating slope of the curve fragment between take off point and maximum.  In the threshold method amplification efficiency is calculated from the slope of standard curve. |
| Average amplification | The average amplification is the average of non-outlier amplification efficiencies calculated for each sample in the same run. Variation in the average amplification value between runs must be minimal. |
| Relative expression | Difference in expression of gene between sample and calibrator (sample to which all others are compared to).  Different models are known for calculation of relative expression. For model used in this paper see Quantification of gene expression by second derivative method in the online supplement. |
| Fold change | Rate of change of gene expression between samples (e.g. if the difference between mean CTt and maximum sample CTt are 3 cycles, the absolute fold change will be 2^(3)^ = 8 fold or 800%). |
| Normalization factor (NF) | Ratio between the relative expression in individual samples to geometric mean of relative expressions within the whole sample set. |
