# Supplementary material for: PSMB2 and RPL32 are suitable denominators to normalize gene expression profiles in bronchoalveolar cells
Source: BMC Mol Biol. 2008 Jul 31;9:69. doi: 10.1186/1471-2199-9-69 (PMC2529339; doi:10.1186/1471-2199-9-69)
Supplement: Additional file 4 — Figure E1. RNA quality assessment (a representative example) by 2100 Bioanalyzer (Agilent Technologies, Palo Alto, USA). This figure shows typical chromatogram of microcapillary electrophoresis of total RNA preparation of good quality extracted from bronchoalveolar lavage cells. Electropherogram shows 18S and 28S rRNA peaks. FU – Fluorescence units. [file 1471-2199-9-69-S4.doc]

**Figure E1. RNA quality assessment (a representative example) by 2100 Bioanalyzer (Agilent Technologies, Palo Alto, USA).**

This figure shows typical chromatogram of microcapillary electrophoresis of total RNA preparation of good quality extracted from bronchoalveolar lavage cells (RIN 7.9). Electropherogram shows 18S and 28S rRNA peaks. FU - Fluorescence units.

**Figure E1.**
