# Supplementary material for: PSMB2 and RPL32 are suitable denominators to normalize gene expression profiles in bronchoalveolar cells
Source: BMC Mol Biol. 2008 Jul 31;9:69. doi: 10.1186/1471-2199-9-69 (PMC2529339; doi:10.1186/1471-2199-9-69)
Supplement: Additional file 5 — Figure E2. Expression levels of ten housekeeping genes in bronchoalveolar cells from sarcoidosis patients and normal subjects from the 2nd cohort. Expression levels of ten housekeeping genes in CTt values in bronchoalveolar cells from sarcoidosis patients (n = 63) a normal subjects (n = 17). The data are presented as means (columns) ± SD (errorbars). White columns represent the control group, dark columns sarcoidosis patients. [file 1471-2199-9-69-S5.doc]

**Figure E2. Expression levels of ten housekeeping genes in bronchoalveolar cells from sarcoidosis patients and control subjects from the 2nd cohort.**


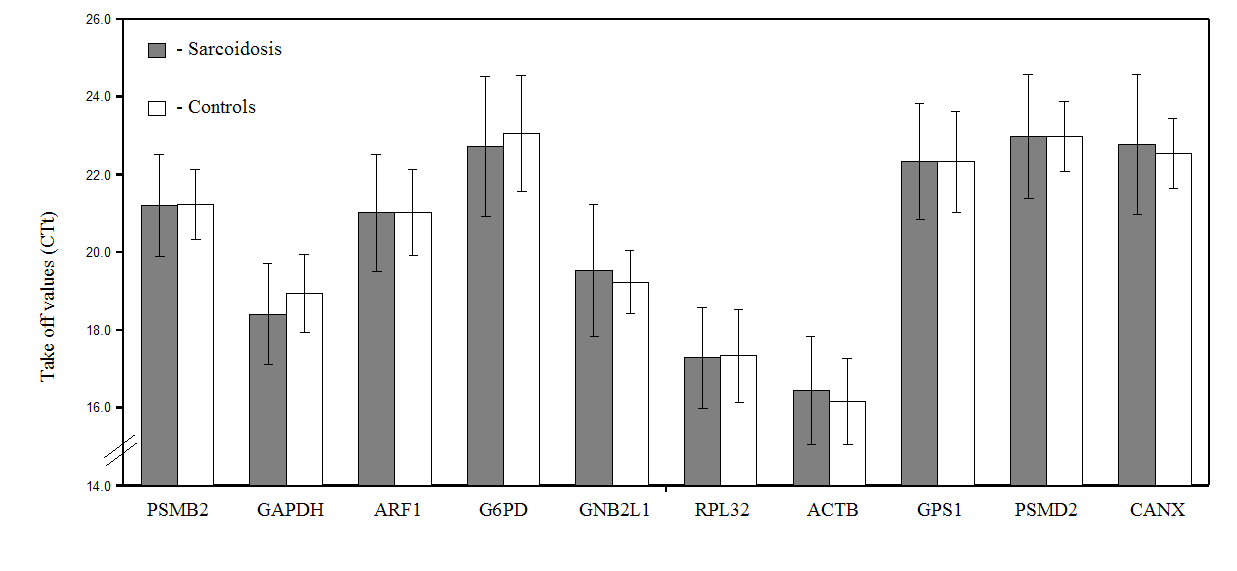
Expression levels of ten housekeeping genes in CTt values in bronchoalveolar cells from sarcoidosis patients (n=63) a normal subjects (n=17). The data are presented as means (columns) ± SD (errorbars). White columns represent the control group, dark columns sarcoidosis patients.
